# Antiproliferative Effects of Methanolic Extracts of *Cryptocarya concinna* Hance Roots on Oral Cancer Ca9-22 and CAL 27 Cell Lines Involving Apoptosis, ROS Induction, and Mitochondrial Depolarization

**DOI:** 10.1155/2014/180462

**Published:** 2014-10-15

**Authors:** Hurng-Wern Huang, Yi-An Chung, Hsun-Shuo Chang, Jen-Yang Tang, Ih-Sheng Chen, Hsueh-Wei Chang

**Affiliations:** ^1^Institute of Biomedical Science, National Sun Yat-Sen University, Kaohsiung 80424, Taiwan; ^2^Graduate Institute of Natural Products, College of Pharmacy, Kaohsiung Medical University, Kaohsiung 80708, Taiwan; ^3^School of Pharmacy, College of Pharmacy, Kaohsiung Medical University, Kaohsiung 80708, Taiwan; ^4^Department of Radiation Oncology, Faculty of Medicine, College of Medicine, Kaohsiung Medical University, Kaohsiung 80708, Taiwan; ^5^Department of Radiation Oncology, Kaohsiung Medical University Hospital, Kaohsiung 80708, Taiwan; ^6^Cancer Center, Translational Research Center, Kaohsiung Medical University Hospital, Kaohsiung Medical University, Kaohsiung 80708, Taiwan; ^7^Institute of Medical Science and Technology, National Sun Yat-sen University, Kaohsiung 80424, Taiwan; ^8^Department of Biomedical Science and Environmental Biology, Kaohsiung Medical University, Kaohsiung 80708, Taiwan

## Abstract

*Cryptocarya*-derived natural products were reported to have several biological effects such as the antiproliferation of some cancers. The possible antioral cancer effect of *Cryptocarya*-derived substances was little addressed as yet. In this study, we firstly used the methanolic extracts of *C. concinna* Hance roots (MECCrt) to evaluate its potential function in antioral cancer bioactivity. We found that MECCrt significantly reduced cell viability of two oral cancer Ca9-22 and CAL 27 cell lines in dose-responsive manners (*P* < 0.01). The percentages of sub-G1 phase and annexin V-positive of MECCrt-treated Ca9-22 and CAL 27 cell lines significantly accumulated (*P* < 0.01) in a dose-responsive manner as evidenced by flow cytometry. These apoptotic effects were associated with the findings that intracellular ROS generation was induced in MECCrt-treated Ca9-22 and CAL 27 cell lines in dose-responsive and time-dependent manners (*P* < 0.01). In a dose-responsive manner, MECCrt also significantly reduced the mitochondrial membrane potential in these two cell lines (*P* < 0.01–0.05). In conclusion, we demonstrated that MECCrt may have antiproliferative potential against oral cancer cells involving apoptosis, ROS generation, and mitochondria membrane depolarization.

## 1. Introduction

Oral squamous cell carcinoma (OSCC) is a type of cancer that frequently occurs in oral cavity. Although it is comparatively easy to clinically inspect by a dentist or to detect by some OSCC tumor markers [1, 2], this carcinoma is usually ignored by patients especially for the early stage. Subsequently, OSCC is frequently diagnosed at advanced stages which then lead to high mortality [3]. Therefore, the drug development of antioral cancer is still necessary and remains to be a challenge.

Natural products have improved the drug discovery for anticancer therapy [4]. For example, some anticancer drugs derived from natural products were approved by the United States Food and Drug Administration [5]. In basic researches, natural products with antioral cancer effects have increasingly being reported. This holds for the ethanolic and methanolic extracts of red alga* Gracilaria tenuistipitata* [6, 7], crude extracts of* Selaginella tamariscina* (oriental medicinal herb) [8], green tea [9], goniothalamin from* Goniothalamus* species [10], and 4*β*-hydroxywithanolide E from golden berry [11].

*Cryptocarya* plants (family Lauraceae), comprising about 350 species worldwide, are widely distributed in the tropics and subtropics [12]. This plant group is well known for its common secondary metabolites, containing alkaloids, flavonoids, and *α*-pyrones [12–15]. Several biological effects of* Cryptocarya*-derived natural products have been reported that include anti-dengue virus [16], anti-HIV [17], anti-tuberculosis [18], antiplasmodial [19], antitrypanosomal [20], and anti-inflammatory [21] function.

Anticancer effects of crude extracts of* Cryptocarya* plant are known as well. For example, the ethanolic extracts of fruit and trunk bark of* C. obovata* showed 56% and 23% growth inhibition of human KB cells at 10 *μ*g/mL, respectively [22]. Methanolic extracts of the leaves of* C. griffithiana* provide cytotoxicity forhuman HL60 promyelocytic leukemia cells [23].

Recently, accumulating findings for anticancer effects of pure compounds isolated from* Cryptocarya* plants were reported, especially from methanolic extracts. For example, compounds isolated from methanol extracts of the trunk bark of* C. infectoria* [24], the trunk bark of* C. costata* [25], and the wood of* C. konishii* [26] were reported to be cytotoxic to leukemia cells. Compounds from methanolic extracts of leaves of* C. chinensis*, were shown to be cytotoxic to human lung cancer and glioblastoma cells [27]. These drugs were isolated from the trunk bark, wood, and leaves of* Cryptocarya* sp. However, the bioactivity of the roots of* Cryptocarya* plants remained little investigated, particularly with respect to antioral cancer.

Because* C. concinna* Hance is an evergreen plant commonly distributed in low-altitude forests in Taiwan [28], it is easy to prepare methanolic extracts of the roots of* C. concinna *Hance (namely, for MECCrt). We, therefore, chose two OSCC cell lines, that is, Ca9-22 and CAL 27, to evaluate the possible anticancer function of MECCrt and investigate their drug mechanisms in terms of cell viability, cell cycle distribution, apoptosis, reactive oxygen species (ROS) generation, and mitochondrial depolarization.

## 2. Materials and Methods

### 2.1. Cell Cultures and Methanolic Extracts of * C. concinna*

Two human OSCC cell lines Ca9-22 and CAL 27, purchased from the Cell Bank, RIKEN BioResource Center (Tsukuba, Japan) and the American Type Culture Collection (ATCC; Virginia, USA), respectively, were incubated in DMEM/F12 (3 : 2) medium (Gibco, Grand Island, NY, USA) supplemented with 10% fetal bovine serum (Gibco), 100 U/mL penicillin, 100 *μ*g/mL streptomycin, and 0.03% glutamine. These two cell lines were humidly incubated at 37°C with 5% CO_2_ in the humid atmosphere.

*C. concinna* was identified by one of the authors (Ih-Sheng Chen) and its roots were collected at Mudan, Pingtung County, Taiwan, in May 2004. A voucher specimen (Chen 6153) has been deposited in the Herbarium of the School of Pharmacy, College of Pharmacy, Kaohsiung Medical University. The dried roots of* C. concinna* were processed by slicing and cold methanol-extraction for three times at room temperature. Finally, the solution was evaporated under reduced pressure to yield the methanolic extract (MECCrt). MECCrt was stored at −20°C and dissolved in dimethyl sulfoxide (DMSO) before treatment.

### 2.2. Cell Viability

Cell viability was measured by the CellTiter 96 AQueous one solution cell proliferation assay (MTS) (Promega Corporation, Madison, WI, USA) as previously described [11]. Ca9-22 and CAL 27 cell lines were seeded at a density of 1 × 10^5^ and 2 × 10^5^ cells per well in a 6-well plate, respectively. After plating for 24 h, these cells were incubated with different concentrations of MECCrt for 24 h and finally subjected to a MTS assay applying an ELISA reader at 490 nm.

### 2.3. Cell Cycle Progression and Sub-G1 Population

Propidium iodide (PI, Sigma, St. Louis, MO, USA) was added to stain the cellular DNA content [29]. In brief, 3 × 10^5^ cells per well in 6 well plates were plated for 24 h and then treated with vehicle (DMSO; 1 *μ*L/2 mL culture medium) as a control or 5, 10, 15, 20, and 25 *μ*g/mL of MECCrt for 24 h. After exposure termination, cells were centrifuged, washed twice with PBS, fixed overnight with 70% ethanol, and centrifuged. Subsequently, the cell pellets were resuspended in 50 *μ*g/mL PI reagent and stand for 30 min at 37°C in darkness. Cell cycle distribution was evaluated by a flow cytometer (BD Accuri C6; Becton-Dickinson, Mansfield, MA, USA) and a BD Accuri C6 Software (version 1.0.264).

### 2.4. Apoptosis

To validate apoptosis in MECCrt-treated oral cancer cells, annexin V (Strong Biotect Corporation, Taipei, Taiwan) [30]/PI (Sigma, St Louis, MO, USA) method was used [31]. Briefly, 3 × 10^5^ cells per well in 6 well plates were plated for 24 h and then treated with vehicle or indicated concentrations of MECCrt for 24 h. Subsequently, apoptotic cells were stained for 30 min with 100 *μ*L binding buffer containing 2 *μ*L of annexin-V-fluorescein isothiocyanate (FITC) stock (0.25 *μ*g/*μ*L) and 2 *μ*L of PI stock (1 mg/mL). Finally, it was suspended with 400 *μ*L PBS for analysis of a flow cytometer (BD Accuri C6; Becton-Dickinson) and its software.

### 2.5. Intracellular ROS

The dye 2′,7′-dichlorodihydrofluorescein diacetate (DCFH-DA) was used to detect ROS by its fluorescence change [7]. Cells at the density of 3 × 10^5^ in 2 mL medium per well in 6 well plates were plated for 24 h. Different concentrations of MECCrt were added to Ca9-22 cells for 6 h and 12 h. After washing with PBS, 100 nM DCFH-DA in PBS was added to cells in 6 well plates at cell culture incubator for 30 min. After trypsinization, PBS washing, and centrifugation, cell pellets were resuspended in 1 mL PBS before analyzing by a flow cytometer (BD Accuri C6; Becton-Dickinson) and its software.

### 2.6. Mitochondrial Membrane Potential

MitoProbe DiOC_2_(3) assay kit (Invitrogen, Eugene, OR, USA) was applied to analyze mitochondrial membrane potential (MMP) as described previously [10]. Briefly, 3 × 10^5^ cells in 2 mL medium per well in 6 well plates were plated for 24 h. After MECCrt treatment, 10 *μ*L of 10 *μ*M DiOC_2_(3) was added per well and incubated in a cell culture incubator for 20 min. After being harvested, cells were washed and resuspended in 1 mL PBS for analysis using a flow cytometer (BD Accuri C6; Becton-Dickinson) and its software.

### 2.7. Statistical Analysis

The significance of differences was determined by Student's *t*-test compared with the test data with the vehicle controls. Data are expressed as means ± SDs.

## 3. Results

### 3.1. Antiproliferation in MECCrt-Treated Two Oral Cancer Cell Lines

**Figure 1:**
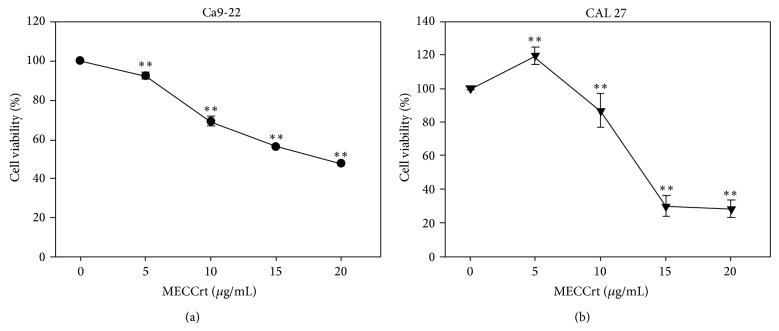
Cell viability of two oral cancer cells was inhibited by MECCrt. Oral cancer Ca9-22 and CAL 27 cell lines were treated with various concentrations of MECCrt (0, 5, 10, 15, and 20 *μ*g/mL) for 24 h. The cell viability was measured by the MTS assay. Data, means ± SDs (*n* = 18). ^**^ *P* < 0.01 against vehicle.

Based on MTS assay (Figure 1), the relative cell viability (%) of oral cancer Ca9-22 cells at indicated concentrations of MECCrt (0, 5, 10, 15, and 20 *μ*g/mL) was 100.0 ± 0.7, 93.3 ± 2.3, 71.4 ± 3.0, 57.6 ± 1.6, and 48.4 ± 1.2, after 24 h, respectively. The relative cell viability (%) of CAL 27 cells at indicated concentrations of MECCrt (0, 5, 10, 15, and 20 *μ*g/mL) was100.0 ± 0.8,119.3 ± 4.9, 86.9 ± 10.0, 29.8 ± 6.2, and 28.4 ± 5.5, respectively. The MTS-based cell viabilities of MECCrt-treated two oral cancer Ca9-22 and CAL 27 cell lines significantly reduced in a dose-responsive manner (*P* < 0.01 compared to the vehicle).

### 3.2. Sub-G1 Population in MECCrt-Treated Two Oral Cancer Cell Lines

**Figure 2:**
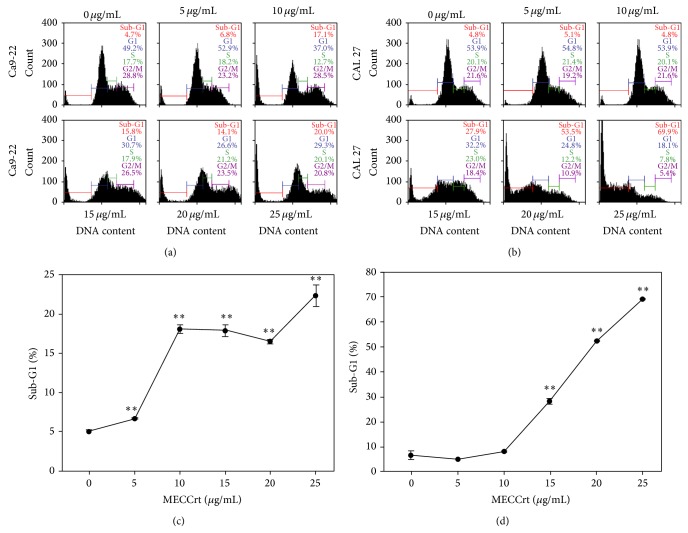
The sub-G1 accumulation of two oral cancer cells was induced by MECCrt. Oral cancer Ca9-22 and CAL 27 cell lines were treated with 0, 5, 10, 15, 20, and 25 *μ*g/mL of MECCrt for 24 h. ((a) and (b)) Representative cell cycle distribution profiles of flow cytometry for MECCrt-treated oral cancer Ca9-22 and CAL 27 cells and vehicles at 24 h, respectively. ((c) and (d)) Statistics analyses for the percentages of sub-G1 population in (a) and (b), respectively. Data, means ± SDs (*n* = 3). ^**^ *P* < 0.01 against vehicle.

The MECCrt-treated effects of cell cycle distribution profiles are demonstrated in Figure 2(a). After MECCrt treatment (Figure 2(b)), the sub-G1 populations (%) of MECCrt- (0, 5, 10, 15, 20, and 25 *μ*g/mL) treated oral cancer Ca9-22 cells were 5.0 ± 0.3, 6.7 ± 0.1, 18.1 ± 0.6, 17.9 ± 0.7, 16.5 ± 0.3, and 22.4 ± 1.3 and those of MECCrt-treated CAL 27 cells were 6.7 ± 1.7, 5.1 ± 1.1, 7.9 ± 0.1, 28.3 ± 1.0, 52.6 ± 0.2, and 69.1 ± 0.1, respectively. These sub-G1 changes significantly accumulated in a dose-responsive manner (*P* < 0.01).

### 3.3. Apoptosis of MECCrt-Treated Two Oral Cancer Cell Lines

**Figure 3:**
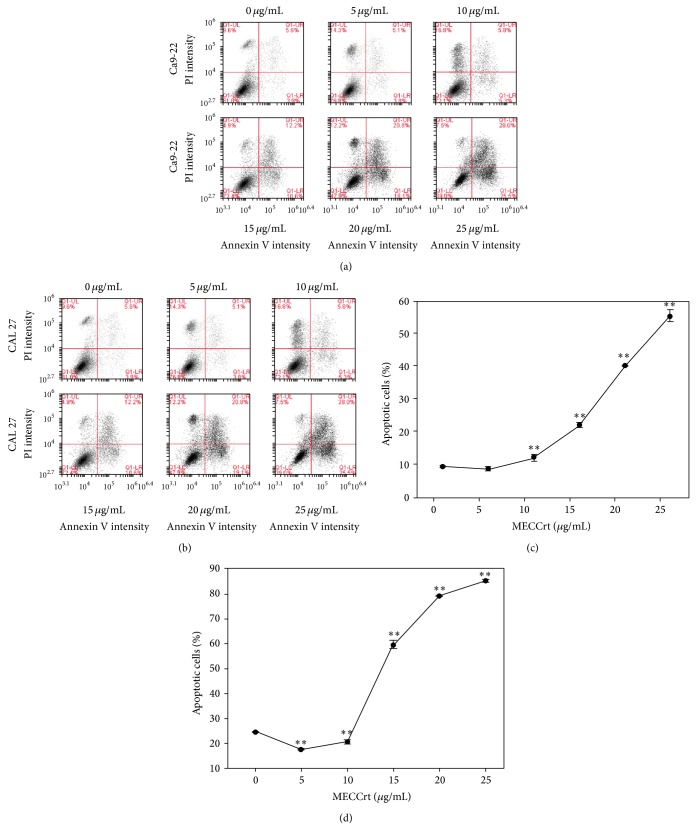
Apoptosis of two oral cancer cells was induced by MECCrt. Oral cancer Ca9-22 and CAL 27 cell lines were treated with 0–25 *μ*g/mL of MECCrt for 24 h. ((a) and (b)) Representative results of annexin V/PI double staining of flow cytometry for MECCrt-treated oral cancer Ca9-22 and CAL 27 cell lines and vehicle controls at 24 h, respectively. ((c) and (d)) Quantification analysis of apoptosis for MECCrt-treated oral cancer Ca9-22 and CAL 27 cell lines in (a) and (b), respectively. Data, means ± SDs (*n* = 3). ^**^ *P* < 0.01 against vehicle.

To validate the possible outcome of apoptosis in MECCrt-induced sub-G1 accumulation of these two oral cancer cells, annexin V/PI profiles of flow cytometry were generated (Figure 3(a)). In Figure 3(b), the percentages of annexin V-positive intensities for MECCrt (0, 5, 10, 15, 20, and 25 *μ*g/mL) treatment of Ca9-22 cells were 9.3 ± 0.2, 8.6 ± 0.3, 11.7 ± 0.9, 22.3 ± 0.8, 40.0 ± 0.2, and 54.4 ± 1.7 and those of MECCrt-treated CAL 27 cells were 24.8 ± 0.1, 17.5 ± 0.3, 20.7 ± 0.4, 59.8 ± 1.7, 79.4 ± 0.2, and 85.3 ± 0.5, respectively. Accordingly, MECCrt treatments significantly increased in annexin V-positive intensities of two oral cancer Ca9-22 and CAL 27 cell lines in a dose-responsive manner (*P* < 0.01).

### 3.4. ROS Generation in MECCrt-Treated Two Oral Cancer Cell Lines

**Figure 4:**
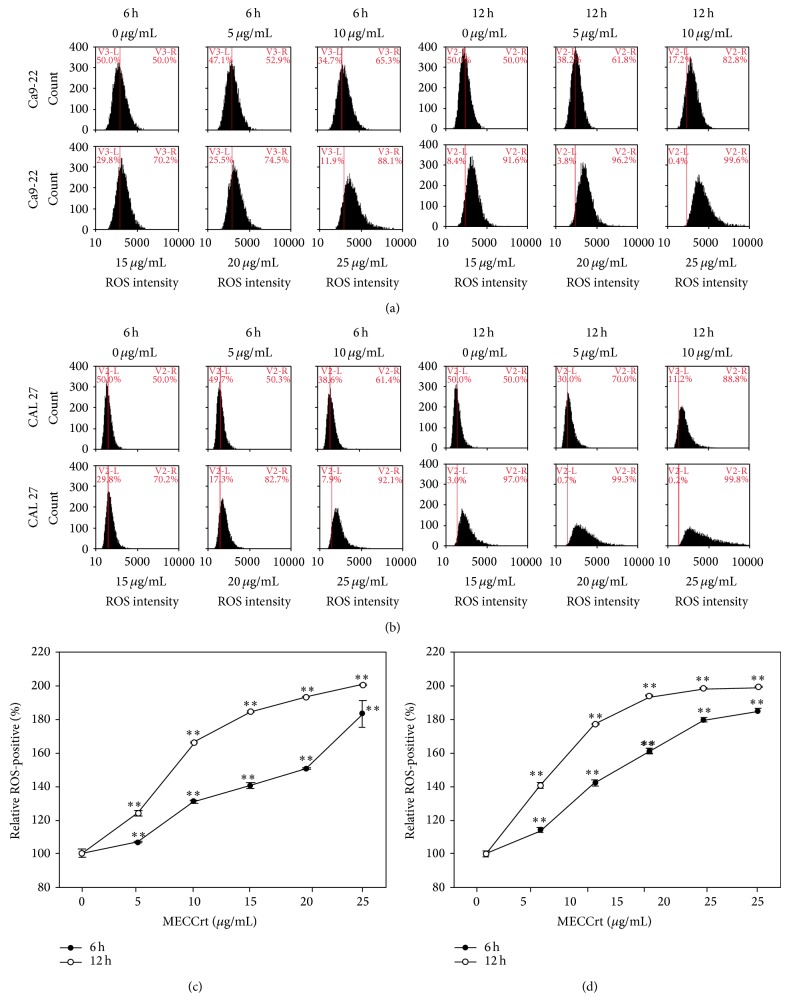
Reactive oxygen species (ROS) generation of two oral cancer cell lines was induced by MECCrt. Oral cancer Ca9-22 and CAL 27 cell lines were treated with different concentrations (0–25 *μ*g/mL) of MECCrt for 6 and 12 h. ((a), (b)) Representative ROS profiles of flow cytometry for MECCrt-treated oral cancer Ca9-22 and CAL 27 cell lines. ((c) and (d)) Statistics analysis of relative ROS intensity in (a) and (b), respectively. Data, means ± SDs (*n* = 3). ^**^ *P* < 0.01 against vehicle.

To validate the role of ROS in the MECCrt-induced apoptosis of two oral cancer cell lines, a DCFH-DA assay of flow cytometry was chosen. Figures 4(a) and 4(b) show the relative ROS-positive staining (%) of two oral cancer Ca9-22 and CAL 27 cell lines for the different concentrations of MECCrt treatment for 6 and 12 h incubation. After MECCrt treatment for 6 h, the relative ROS-positive staining (%) of 0, 5, 10, 15, 20, and 25 *μ*g/mL MECCrt-treated Ca9-22 cells was 100.0 ± 1.8, 106.7 ± 0.4, 131.2 ± 0.8, 140.6 ± 1.6, 150.6 ± 0.3, and 183.1 ± 7.8 and that of MECCrt-treated CAL 27 cells was 100.0 ± 0.8, 114.1 ± 1.4, 142.3 ± 1.5, 161.1 ± 0.7, 179.8 ± 1.1, and 185.1 ± 1.4, respectively. After MECCrt treatment for 12 h, the relative ROS-positive staining (%) of 0, 5, 10, 15, 20, and 25 *μ*g/mL MECCrt-treated Ca9-22 cells was 100.0 ± 2.3, 124.1 ± 0.4, 166.4 ± 1.2, 184.8 ± 0.6, 193.3 ± 0.3, and 200.5 ± 0.0 and that of MECCrt-treated CAL 27 cells was 100.0 ± 0.9, 140.7 ± 1.4, 177.1 ± 0.3, 193.8 ± 0.1, 198.6 ± 0.1, and 199.4 ± 0.1, respectively. Accordingly, MECCrt treatments significantly increased in both dose-responsive and time-dependent manners in these two oral cancer cell lines (*P* < 0.05) (Figures 4(c) and 4(d)).

### 3.5. MMP Depolarization in MECCrt-Treated Two Oral Cancer Cell Lines

**Figure 5:**
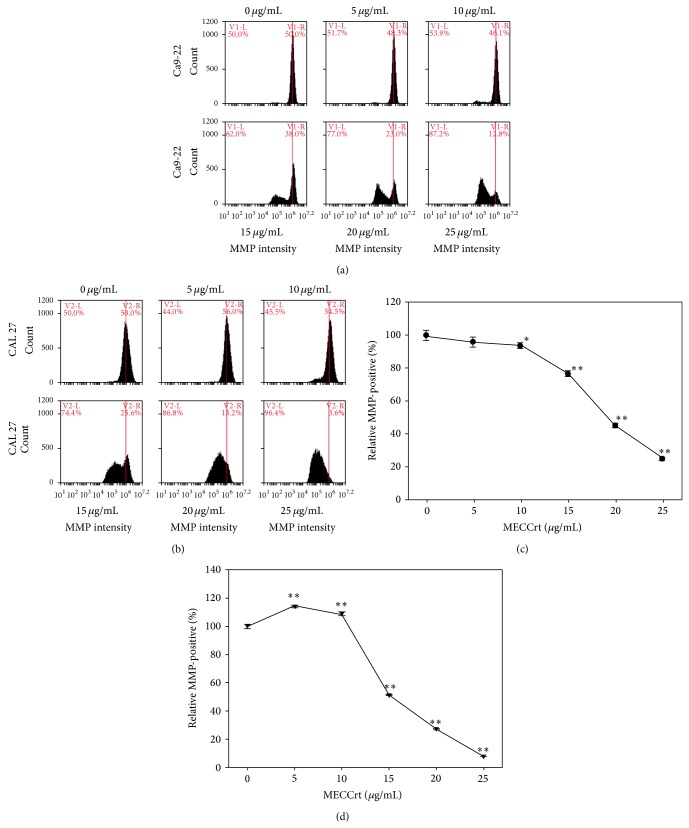
Depolarization of mitochondrial membrane potential (MMP) of Ca9-22 and CAL 27 oral cancer cell lines was induced by MECCrt. Oral cancer Ca9-22 and CAL 27 cell lines were treated with different concentrations (0–25 *μ*g/mL) of MECCrt for 24 h. ((a), (b)) Representative MMP profiles of flow cytometry for MECCrt-treated oral cancer Ca9-22 and CAL 27 cells. ((c) and (d)) Quantification analysis of relative MMP intensity in (a) and (b), respectively. Data, means ± SDs (*n* = 3). ^*^ *P* < 0.05 and ^**^ *P* < 0.01 against vehicle.

Figures 5(a) and 5(b) show the MMP profiles of DiOC_2_(3)-positive intensities for the vehicle and MECCrt-treated oral cancer cell lines in 24-hour treatments. Treated with MECCrt (0, 5, 10, 15, 20, and 25 *μ*g/mL) for 24 h, the DiOC_2_(3)-positive (%) intensities of Ca9-22 cells were 100.0 ± 2.9, 96.1 ± 2.4, 94.0 ± 1.6, 76.8 ± 1.4, 45.6 ± 1.4, and 25.0 ± 1.1, respectively. Similarly, the percentages of DiOC_2_(3)-positive (%) intensities of MECCrt-treated CAL 27 cells were 100.0 ± 1.5, 114.2 ± 0.7, 108.9 ± 1.3, 51.3 ± 0.5, 27.3 ± 0.5, and 7.5 ± 0.4, respectively. Accordingly, MECCrt significantly reduced DiOC_2_(3)-positive intensities of two oral cancer Ca9-22 and CAL 27 cell lines in a dose-responsive manner (*P* < 0.01–0.05).

## 4. Discussion

We discovered for the first time that methanolic extracts of the roots of* C. concinna* Hance have an antiproliferative effect on two oral cancer cell lines. The proliferation inhibiting function of MECCrt against oral cancer Ca9-22 and CAL 27 cell lines was dose-responsive (Figure 1).

The anticancer effects for other* Cryptocarya*-derived compounds from methanolic extracts of nonroot parts have been reported earlier. For example, for murine leukemia P-388 cells, the IC_50_ values of 2′,4′-dihydroxy-5′,6′-dimethoxychalcone, and isodidymocarpin, isolated from tree bark of* C. costata*, were 5.7 and 11.1 *μ*M [25] and IC_50_ values of the chalcone derivative (desmethylinfectocaryone) and phenolic compound (infectocaryone), isolated from wood of* C. konishii*, were 2.17 and 0.8 *μ*M [26] at 48 h, respectively. For compounds from leaves of* C. chinensis*, the IC_50_ values of infectocaryone and cryptocaryanone A were at the *μ*M level for human lung cancer NCI-H460 cells and glioblastoma SF-268 cells [27]. These* Cryptocarya*-derived compounds from methanolic extracts of nonroot parts showed the IC_50_ values ranging from 0.8 to 11 *μ*M. This is close to our preliminary result that the IC_50_ of the clinical anticancer drug cisplatin at 24 h treatment in oral cancer Ca9-22 cells is 3.06 *μ*g/mL (10.2 *μ*M) (data not shown). In the present study, the IC_50_ values of the MECCrt in oral cancer Ca9-22 and CAL 27 cell lines at 24 h were 18.67 and 13.22 *μ*g/mL, respectively. Although the IC_50_ values of the MECCrt were about 3-4 folds of cisplatin for oral cancer cells, its crude extract nature has to be concerned. Therefore, it is warranted to further investigate the particular bioactive components that are included in the methanolic extracts of* Cryptocarya concinna* Hance roots.

Moreover, the anticancer effect for trunk bark of* C. infectoria*-derived methanol extracts was reported to be cytotoxic to KB cells [24]. KB cells were regarded as oral epidermal carcinoma, however, it was recently validated to have marker chromosomes and DNA finger printings of human cervical cancer HeLa cells (http://www.ncbi.nlm.nih.gov/mesh?Db=mesh&term=KB+Cells) [32]. Accordingly, the anticancer effect of oral cancer by the bioactive compounds from* Cryptocarya* plant remains unclear. Conversely, we here demonstrate the antioral cancer effect of methanolic extracts of a* Cryptocarya* species for the first time, using two OSCC cell lines Ca9-22 and CAL 27.

In several anticancer drugs [6, 7, 10, 11, 33–36], ROS generation is one of the common strategies to inhibit cancer cell proliferation. ROS plays a vital role in early stages of apoptosis [37] and leads to MMP depolarization [38, 39]. Escaping apoptosis is demonstrated to be involved in the drug resistance of cancer cells [40, 41]. To enhance apoptotic induction of anticancer drugs may interfere the drug resistance if there. In the present study, we observed that apoptosis was inducible by MECCrt in two OSCC cell lines as it was demonstrated by sub-G1 monitoring and annexin V/PI assay. We also found that MECCrt significantly induced the ROS level and reduced the MMP level in two oral cancer cell lines in dose-responsive ways. These findings suggest that oxidative stress may be involved in the MECCrt-induced antiproliferative effect in two oral cancer Ca9-22 and CAL 27 cell lines. However, the role of oxidative stress in MECCrt need to be further examined by the ROS scavenger such as N-acetylcysteine [42] to confirm if raised ROS has played a critical role in the process of apoptosis. Furthermore, the ROS may generate nonapoptotic effect like autophagy described in literature [43, 44]. Therefore, it was warranted to further investigate the role of autophagy in MECCrt-treated oral cancer cell lines in future.

## 5. Conclusions

We demonstrated the antiproliferative and apoptotic effects of MECCrt through ROS generation and mitochondrial depolarization in two OSCC cell lines. Therefore, these results suggest that MECCrt has anticancer potential for oral cancer therapy.
